# *In-situ* understanding on the formation of fibrillar morphology in green solvent processed all-polymer solar cells

**DOI:** 10.1093/nsr/nwae384

**Published:** 2024-11-04

**Authors:** Ruijie Ma, Hongxiang Li, Top Archie Dela Peña, Heng Wang, Cenqi Yan, Pei Cheng, Jiaying Wu, Gang Li

**Affiliations:** Department of Electrical and Electronic Engineering, Research Institute for Smart Energy (RISE), Photonic Research Institute (PRI), The Hong Kong Polytechnic University, Hong Kong 999077, China; College of Polymer Science and Engineering, State Key Laboratory of Polymer Materials Engineering, Sichuan University, Chengdu 610040, China; Function Hub, Advanced Materials Thrust, The Hong Kong University of Science and Technology, Guangzhou 511400, China; College of Polymer Science and Engineering, State Key Laboratory of Polymer Materials Engineering, Sichuan University, Chengdu 610040, China; College of Polymer Science and Engineering, State Key Laboratory of Polymer Materials Engineering, Sichuan University, Chengdu 610040, China; College of Polymer Science and Engineering, State Key Laboratory of Polymer Materials Engineering, Sichuan University, Chengdu 610040, China; Function Hub, Advanced Materials Thrust, The Hong Kong University of Science and Technology, Guangzhou 511400, China; Department of Electrical and Electronic Engineering, Research Institute for Smart Energy (RISE), Photonic Research Institute (PRI), The Hong Kong Polytechnic University, Hong Kong 999077, China

**Keywords:** all-polymer solar cells, *in-situ* morphology screening, naphthalene-based solid additives, phase segregation

## Abstract

Solid additive engineering has been intensively explored on morphology tuning for highly efficient all-polymer solar cells (all-PSCs), a promising photovoltaic technology towards multi-scenario application. Although the nano-fibrillar network of the active layer induced by additive treatment is confirmed as the key factor for power conversion efficiency (PCE) of all-PSCs, its formation mechanism is not clearly revealed, for lack of precise and convincing real-time observation of crystallization and phase separation during the liquid-to-solid transition process of spin-coating. Herein we report an *in-situ* grazing incidence wide-angle/small-angle X-ray scattering (GIWAXS/GISAXS) screening that reveals the fact that naphthalene derived solid additives can suppress the aggregation of the polymer acceptor (PY-IT) at the beginning stage of spin coating, which provides sufficient time and space for the polymer donor (PM6) to form the fibril structure. Moreover, guided by this knowledge, a ternary all-polymer system is proposed, which achieves cutting-edge level PCEs for both small-area (0.04 cm^2^) (also decent operational stability) and large-area (1 cm^2^) devices.

## Teaser

For the first time *in-situ* GIWAXS and GISAXS technology is applied to spin-coated all-polymer active layers, successfully revealing new mechanisms on solid additive's morphology regulation effect.

## INTRODUCTION

The power conversion efficiency (PCE) of all-polymer solar cell (all-PSC) has been found dramatically affected by the use of additive(s), which assists the development of device performance when exceeding 19% [1–5]. It has been frequently confirmed by reports that naphthalene derivatives are one of the most effective types [6–13]. On the other hand, the solidified additives have emerged as the hope of promoting PCEs for various blend systems [14–25]. Logically, solid additives derived from naphthalene are confirmed as yielding the best performances on all-PSCs [20,26,27].

Though the naphthalene functionalized solid additives have been revealed as crucial to realizing donor-acceptor bi-continuous fibrillar networks, the reason of the formation of such morphology lacks a detailed observation and analysis. Apart from post treatment, thermal annealing (TA), solid additive's effect on the film formation process has been rarely investigated using suitable methods. In comparison, the liquid to solid transition for active layer deposition includes nucleation, crystallization, and aggregation, which is more complicated and dominant than TA treatment. Thus, a clearer observation on this process is required to reveal the mechanism of achieving performance favorable donor-acceptor phase distribution through advanced technologies, such as operando GIWAXS and GISAXS [28–35].

In this work, we investigate a series of functionalized naphthalene solid additives: 2-chloronaphthalene (2-CN), 2-methylnaphthalene (2-MN), 2-methoxynaphthalene (2-oMN), and 2-methylthionaphthalene (2-sMN). Based on them, a complete optimization of additive concentration on PM6:PY-IT precursor [36], has been carried out to screen the device performance variation. Consequently, the devices treated by additives of suitable concentrations demonstrate similarly improved PCEs (from <14% to >17%), mainly attributed to a significantly enhanced fill factor (*FF*). The delayed recombination kinetics led by additive treatment is found realized by satisfactorily promoting charge mobilities. Further *ex-situ* morphological characterizations reveal that this improvement is realized by induced formation of nano-fibrillar network structures, while additive-free film has a low-continuity phase segregated structure. Interestingly, the real time gained GIWAXS and GISAXS data during the spin coating process tell of an uncommon conclusion: the crystallization process (nucleation and aggregation) and final-state crystallinity are barely correlated to variations in charge behavior, while the solidified film's domain length scale matters more. Moreover, the prerequisite of realizing a nano-fibrillar structure has been confirmed here, which suppresses the aggregation behavior of PY-IT (polymer acceptor) at the early stage, leading to a preformed fibrillar network generated by PM6 for PY-IT's domain growth at a later time. Otherwise, fast domain expansion of PY-IT at the beginning would result in an undesirable competition with PM6’s aggregation that impedes the construction of nanofiber structures. In addition, insufficient additives lead to non-fully suppressed PY-IT aggregation, thus imperfect phase distribution, while an overdose of additives causes excessive liquid-liquid phase separation, which once again yields oversized pure domains. Based on these understandings, we further explore the device performance potential by well-developed ternary blends on both small-area and large-area (1 cm^2^) devices. The obtained 19.27% and 17.66% efficiencies are among the highest values of all-PSCs processed by green solvents.

## RESULTS AND DISCUSSION

**Figure 1.**
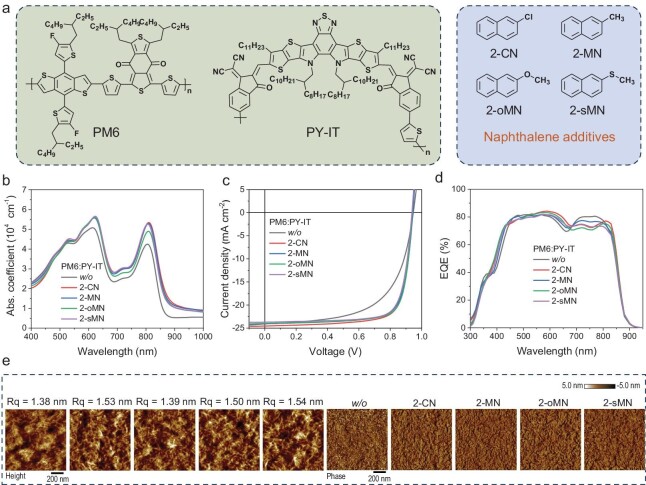
(a) Chemical structures of PM6, PY-IT, and naphthalene derivatives. (b) The blend film absorption profiles. (c) *J−V* characteristics. (d) EQE spectra. (e) AFM height and phase images.

The chemical structures of active layer materials, PM6 and PY-IT, four additives: 2-CN, 2-MN, 2-oMN, and 2-sMN are illustrated in Fig. 1a. Among them, 2-CN and 2-oMN have been reported effective in promoting all-PSC's device performance [20,26,27]. It is noteworthy that not only naphthalene-based solid additives promote the performance of the PM6-PY-IT–based photoactive layer, some other materials such as DIB are reported effective as well [1]. Here we only focus on naphthalene derivatives to make a systematic conclusion. Therefore, considering the highly similar structures, 2-MN and 2-sMN, are supposed to be useful as well. Subsequently, the ultraviolet-visible (UV-vis) absorption spectra are measured and put in normalized format (Fig. 1b). The results indicate that additive treatment would lead to significantly tuned PM6 aggregation in film, while equally on the polymer acceptor's relative aggregation intensity, which can be further explored.

Subsequently, a direct comparison and optimization of naphthalene additives on all-PSC performance modulation is implemented by fabricating a series of devices of ITO/PEDOT:PSS/PM6:PY-IT/PFN-Br-MA/Ag structures [37,38]. The related current density versus voltage (*J−V*) characteristics are shown in Fig. 1c and Fig. S1, with the extracted photovoltaic parameters displayed in Table S1. The normal distribution of PCE values based on 10 independent devices are depicted in Fig. S2. The results show that appropriate ratio of additive incorporation is of great importance to realizing simultaneously decent *J*_SC_ and *FF* for high PCE. Low content additive leads to unsatisfactory *FF* though promoted *J*_SC_, and too large an introduction results in high *FF* yet significantly reduced *J*_SC_, implying insufficient phase segregation or fibrillization for the former, and overly pure phase expansion though completely formed fibrillar structure for the latter. Consequently, only suitably optimized content for all four additive-based devices yield desirable PCE.

To ensure the accuracy of measured efficiencies, the external quantum efficiency (EQE) spectra of all systems are presented in Fig. 1d and Fig. S3, whose integrated current density (*J*_cal_) values are demonstrated in Table S1, as well. The measurement error is therefore within 3%, a reliable index. Besides, the EQE spectra indicate naphthalene derivatives rarely change the bandgap of the PM6:PY-IT system. Considering the generally similar *V*_OC_ values, there leaves limited meaning to evaluate the energy loss.

The morphology feature of optimized additive treated active layers and the as-cast counterpart is investigated by commonly used *ex-situ* technologies, here atomic force microscopy (AFM) and grazing-incidence wide-angle X-ray scattering (GIWAXS) experiments [39–42]. The AFM height and phase images are demonstrated in Fig. 1e, in which the untreated film possesses incomplete construction while the nano-scale fiber formed network can be observed from all optimal content additive processed films. On this point, it would be easy to conclude that additive induced fibrillization in film offers improved charge transport and well-kept charge generation.

A special note should be emphasized that such device parameter variation and *ex-situ* morphology characteristic transitions are self-explanatory to why the PM6:PY-IT blend is chosen. Compared to polymer donor small molecular acceptor systems, that can form fibrillar networks without the introduction of additives, for instance D18:L8-BO/eC9 [43–45], PM6:PY-IT's device performance and morphology features are significantly modulated by solid additives.

The normalized absorption profiles of neat donor and acceptor films are also presented here, where both full wavelength and peak focused range referred spectra are displayed (Fig. S4). It is found that naphthalene-derived solid additives brought a slight red-shift for PM6’s 0–0 and 0–1 peaks, which implies marginally enhanced aggregation, in other words, suppressed backbone relaxation. Meanwhile, the profiles of PY-IT films demonstrate insignificant variation on the main peak region, expect for a very tiny blueshift on the 2-sMN treated one. This phenomenon proves that additive treatment would not change the final aggregation state of PY-IT, though blend film based solar cell performance has been improved tremendously, thereby the key process of morphology tuning must take place at the liquid-to-solid phase transition stage of film formation.

The 2D-GIWAXS patterns are shown in Fig. S5, with line-cut profiles and fitting results in Fig. S6, and Tables S2–S5. The results suggest that naphthalene additives haven't significantly changed the general crystallization motif and crystalline order, which may be contradictory to common feeling. Combined with AFM results, it is now believed that phase separation and domain aggregation behavior plays a dominant role in changing device performance of the PM6:PY-IT system than crystallite property variation. More analyses will be presented later.

**Figure 2.**
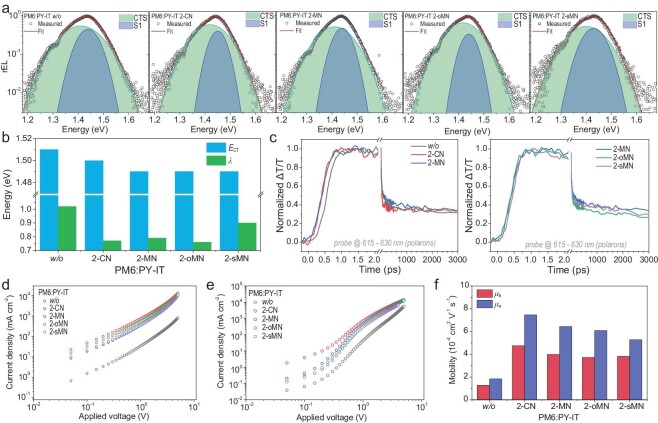
(a) Analyzed reduced EL spectra. (b) Summarized *E*_CT_ and λ values. (c) The interface charge behaviors evaluated by polaron dynamics extracted from the signals of 615 nm to 630 nm region. (d) Hole-only and (e) electron-only device results. (f) Derived hole and electron mobilities.

Despite the low necessity of comparing energy loss, the electroluminescence (EL) test is still instructive, since the relative analysis offers valuable information of charge transfer state energy (*E*_CT_) and reorganization energy (λ), that is helpful to illustrate the energy landscape at the donor/acceptor interface [43]. The obtained EL signals and corresponding fit lines are displayed by Fig. 2a by order, and the calculated *E*_CT_ and λ values are shown in Fig. 2b. Supposing the potentials of material systems are identical as the same active layers that are chosen here, the energy barrier at the interface that concurrently impedes charge generation and recombination has been lowered by treatment with additives. Therefore, in this case, the interface energy landscape is optimized for achieving a more efficient photon capturing, while fibrillization dominates the recombination suppression. Thes phenomenon is then investigated in greater depth via femto-second transient absorption spectroscopy (fs-TAS) technology [44–50]. The resultant 2D contour maps of the blend films are shown in Fig. S7, and the extracted polaron dynamics are visualized by Fig. 2c. It is found that additives can only fasten polaron generation yet remain with undistinguished differences for recombination kinetics. Therefore, in this case, the interface property isn't the dominant factor for device performance.

Accordingly, the pure phase property dominated charge mobility is evaluated using the space charge limited current (SCLC) method [24,51,52]. The hole-only and electron-only *J−V* curves are shown in Fig. 2d and e. Meanwhile, the derived hole mobility (*μ*_h_) and electron mobility (*μ*_e_) values are displayed in Fig. 2f. Apparently evidenced by the mobility change, additive treatment realizes significantly improved charge transport in active layers, consistent to the great increase of *FF* caused by the suppressed free charge recombination.

Accordingly, the donor/acceptor phase fibrillization is the key factor for realizing the high performance of PM6:PY-IT based all-PSCs, which is induced by additives here, is supposed to be attributed to liquid-to-solid transition modulation, since the strong pre-aggregation property of PM6: the preforming of the morphology framework is contributed by it in most cases, thus the post treatment after film solidification would not result in dramatic changes of phase distribution. Therefore, a real time observation on material's crystallization and aggregation during the film formation process is very much needed in order to have a thorough understanding of our case.

**Figure 3.**
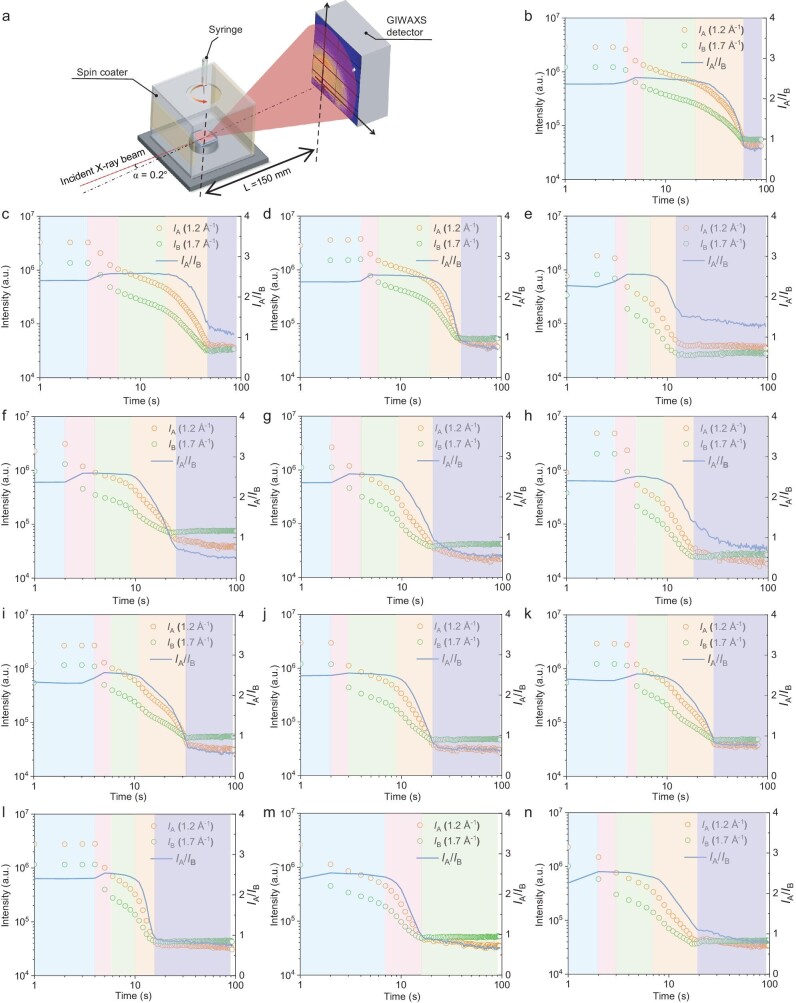
(a) Schematic diagram of *in-situ* GIWAXS measurement. Derived real time crystallization process of PM6:PY-IT films of (b) w/o, (c) 20 mg/mL 2-CN, (d) 30 mg/mL 2-CN, (e) 40 mg/mL 2-CN, (f) 20 mg/mL 2-MN, (g) 30 mg/mL 2-MN, (h) 40 mg/mL 2-MN, (i) 10 mg/mL 2-oMN, (j) 20 mg/mL 2-oMN, (k) 30 mg/mL 2-oMN, (l) 10 mg/mL 2-sMN, (m) 20 mg/mL 2-sMN, (n) 30 mg/mL 2-sMN.

The *in-situ* GIWAXS experiments are then implemented. The dynamic screenings of all films are provided in ‘graphic interchange format’ (GIF) as attached. Because the signal collection results are inevitably interfered with by evaporating solvent, here we only extracted the time-dependent signal intensities of two characteristic peaks for comparison analysis in Fig. 3. The whole liquid-to-solid process is divided into 4 stages: (i) solvent evaporation, where polymers are still at dissolved state; (ii) solvent removing leads to material saturation, in which the materials are nucleating; (iii) based on completed nucleation, crystallization dominates this process; (iv) the films have finished solidification. The key analyzing worthy nucleation and crystallization stages are marked by green and orange backgrounds. To more precisely describe the additive induced crystallization change, a quantitative comparison of nucleation time and crystal growth time is provided in Fig. S8a. Accordingly, the nucleation and crystallization processes are significantly reduced by functionalized naphthalene additives. However, as told by *ex-situ* GIWAXS results, the crystalline features of active layers aren't altered a great deal, though the crystallization periods are greatly different. Hence, film formation process modulation engineering might lead to similar crystal sizes which is dominated by the material's intrinsic properties, i.e. intramolecular and intermolecular interaction. The change of crystalizing process then mainly affects the crystallization-induced phase separation, that is supposed to be demonstrated by *in-situ* GISAXS measurements.

**Figure 4.**
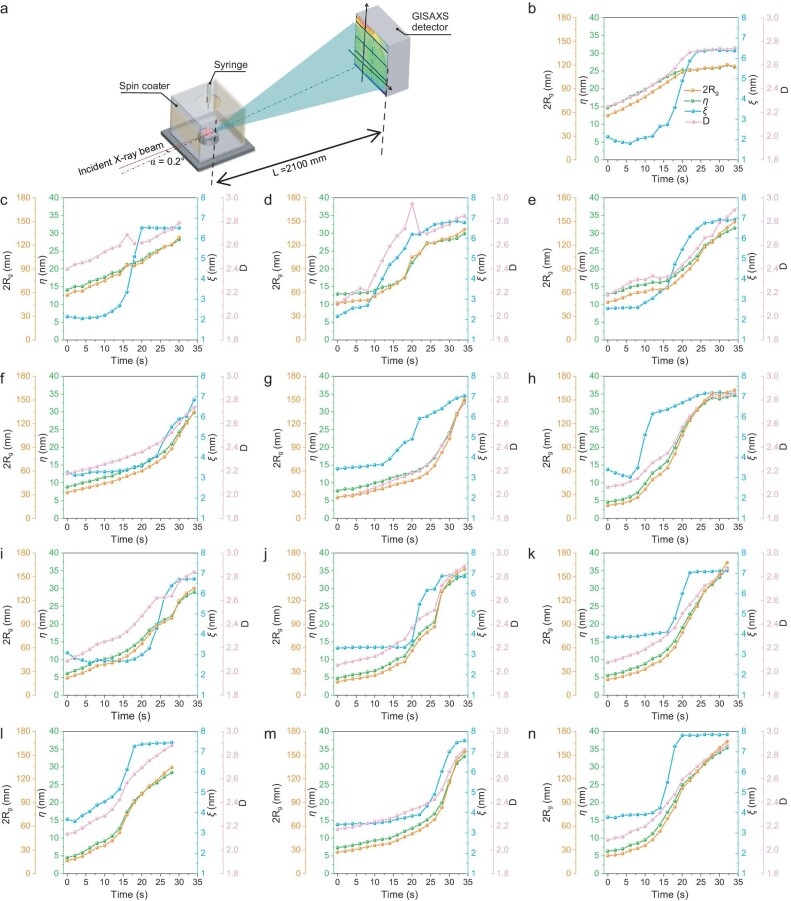
(a) Schematic diagram of *in-situ* GISAXS measurement. Derived real time crystallization process of PM6:PY-IT films of (b) w/o, (c) 20 mg/mL 2-CN, (d) 30 mg/mL 2-CN, (e) 40 mg/mL 2-CN, (f) 20 mg/mL 2-MN, (g) 30 mg/mL 2-MN, (h) 40 mg/mL 2-MN, (i) 10 mg/mL 2-oMN, (j) 20 mg/mL 2-oMN, (k) 30 mg/mL 2-oMN, (l) 10 mg/mL 2-sMN, (m) 20 mg/mL 2-sMN, (n) 30 mg/mL 2-sMN.

Subsequently, the real-time screening of *in-situ* GISAXS was carried out (videos are attached). Figure 4 and Fig. S9 present the experiment set-up, in-plane directional intensity profiles, fit lines, and calculated phase separation parameters. The one order differentiation processed data on phase separation parameters are drawn in Fig. S10. To be specific, the phase separation length scales are described by the following parameters: 2*R*_g_ the pure acceptor phase size, D the phase dimensionality, *η* the correlation length, *ξ* the donor-rich phase scale [53–56]. The real time material aggregation behavior can then be clearly analyzed. The phase length scales of the solidified state are first summarized in Fig. S9b. The general tendency is that applying additives result in significantly increased PY-IT rich domain size, and the PM6 rich phase can be only marginally increased, which can be attributed to the selective solubility of additives in solvent on PY-IT. Then the donor and acceptor rich domain's expansion processes are quantitatively divided into different stages as displayed in Fig. S9c and S9d. PM6’s phase expansion is found to be highly complex, while PY-IT's phase growth demonstrates a more distinguishable trend, that is applying additives realizes slow aggregation behavior. Reflected by the differentiated curves, only untreated blend film shows lower value at the later stage than the initial time for acceptor length scale parameters. In addition, there generally exists ∼5 s stage for the donor phase to quickly expand, and these are all earlier than the acceptor domain's fast growth. This can be understood as suppressing PY-IT aggregation at the beginning stage is beneficial for pre-aggregated PM6 forming a fibrillar framework, because once PY-IT starts to aggregate too early, the uncontrollable phase expansion is going to destroy PM6’s fibrillation process. Insufficient naphthalene-additive treatment leads to less favorable fibrillation, and overdosing would lead to excessive liquid-liquid phase separation. The former cannot achieve a satisfying device *FF*, while the latter would sacrifice donor/acceptor interface area for decent *J*_SC_.

Technically noteworthy, the *in-situ* GIWAXS/GISAXS set-ups are given in Fig. S8, from which we can confirm the fixed alignment and averaged data collection.

**Figure 5.**
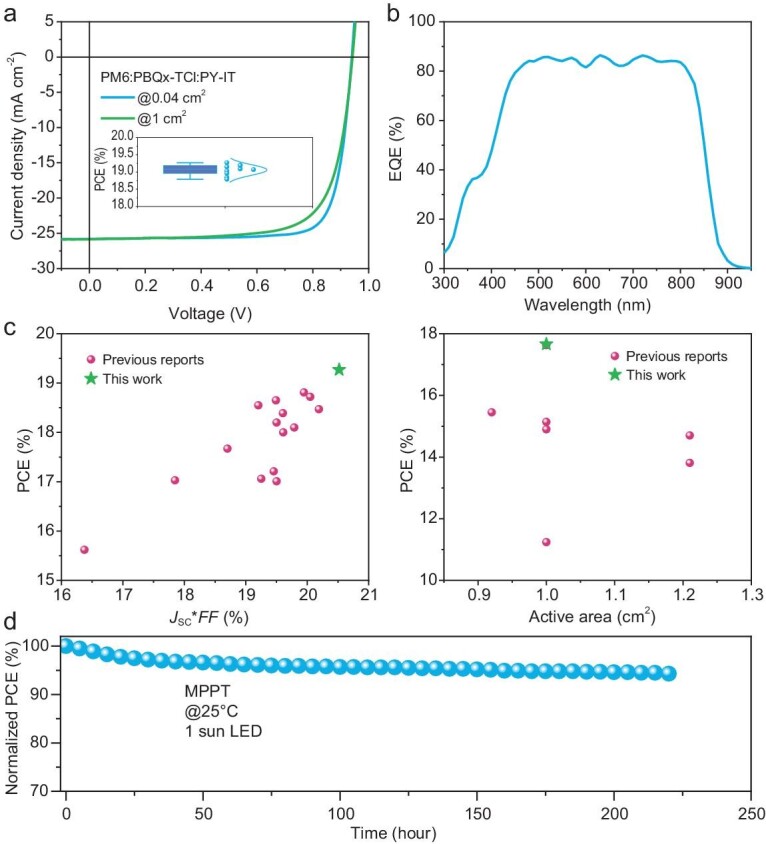
Characterization of PM6:PBQx-TCl:PY-IT (0.5:0.5:1 in weight) solar cells. (a) *J−V* characteristic and normal distribution of PCEs. (b) EQE spectra. (c) Performance summary on green-solvent processed and large-area all-PSCs, respectively. (d) MPPT curve for optimized all-PSCs.

Generally, the fibrillar network is the key factor for achieving high device efficiency for PM6:PY-IT based all-PSCs, which is formed thanks to suppressed acceptor domain growth at the beginning stage. Therefore, further morphology optimization methods could introduce a more aggregated donor polymer into the binary solution to facilitate the completion of fibrillar networks. Herein, we accordingly select PBQx-TCl [57], that's been proven effective in constructing ternary blends with PM6 [58], to explore the probability of promoting the performance of all-PSCs processed from green solvent with green solid additive. Figure 5, thus presents the device performance of a PM6:PBQx-TCl:PY-IT cell fabricated from o-XY with 2-oMN cosolvent (2-oMN is non-toxic, and a component of soap). The *J−V* curves of small-area and large-area devices are plotted in Fig. 5a, and the related parameters are shown in Table 1. The ternary devices yield high efficiencies of 19.27% on 0.041 cm^2^ active area and 17.66% on the 1.00 cm^2^ one, representing the highest level on all-PSCs processed from green solvent or based on large active area by order [59–64]. The EQE spectrum of the optimized device is also provided, which demonstrates the photon utilization improvement effect of introducing PBQx-TCl. Furthermore, the thickness varied *J−V* curves, EQE spectra and related parameters are all provided in Fig. S12 and Table S6 for a more complete comparison. Then, a summary of all-PSC efficiencies on green solvent processed systems and large-area devices of this work and others are displayed in Fig. 5c (details in Tables S7 and S8), which further emphasize this work's engineering significance [65–67]. We will admit that a solar cell with enlarged active area shows lower *FF* due to more severe interface recombination that is driven by the low charge mobility of active materials and imperfect active layer/cathode contact [68]. Last, the device operational stability of the optimized all-PSC is evaluated by maximal output point tracking (MPPT) as plotted in Fig. 5d. It tells that under mild environmental conditions, all-PSCs can yield a decent stability (>90% initial PCE after 200 hour continuous 1-sun illumination).

**Table 1.** Photovoltaic performance.

PM6:PBQx-TCl:PY-IT	*V*_OC_ (V)	*J*_SC_ (mA cm^−2^)	*FF* (%)	PCE (%)
@0.041 cm²	0.939	25.81/24.98	79.5	19.27 (19.06 ± 0.16)
@1.00 cm²	0.941	25.85	72.6	17.66

The average values are based on 10 independent devices.

## CONCLUSION

In summary, here we focus on a well-known all-polymer system PM6:PY-IT, and its classical morphology modulation method of using naphthalene-based additives, using a series of *in-situ* X-ray diffraction characterizations to reveal the real-time film forming mechanism. Consequently, it has been successfully found that additives would suppress the aggregation of PY-IT at the beginning of the liquid-to-solid transition, which provides sufficient time for PM6 to form the nano-fibrillar network. The realized interpenetrating fibrillar morphology simultaneously facilitates efficient charge generation and transport, yielding significantly improved *FF*. As an attempt at device performance promotion, a ternary blend has been examined here, which demonstrates state-of-the-art efficiencies for small-area and large-area all-PSCs processed by green co-solvents.

## Supplementary Material

nwae384_Supplemental_Files
