# Supplementary material for: In-situ understanding on the formation of fibrillar morphology in green solvent processed all-polymer solar cells
Source: Natl Sci Rev. 2024 Nov 4;11(12):nwae384. doi: 10.1093/nsr/nwae384 (PMC11629699; doi:10.1093/nsr/nwae384)
Supplement: nwae384_Supplemental_Files [file nwae384_supplemental_files.zip › Supplementary data.pdf]

Supporting Information:

In-situ Understanding on the Formation of Fibrillar Morphology in Green Solvent Processed All-polymer Solar Cells

Ruijie Ma[1,4]*, Hongxiang Li[2,4]*, Top Archie Dela Peña[3], Heng Wang[2], Cenqi Yan[2], Pei Cheng[2], Jiaying Wu[3], Gang Li[1]*

[1]Department of Electricaland Electronic Engineering, Research Institute for Smart Energy (RISE), Photonic Research Institute (PRI), The Hong Kong Polytechnic University, Hung Hom, Kowloon, Hong Kong 999077, China.

Email: ruijie.ma@polyu.edu.hk; gang.w.li@polyu.edu.hk

[2]College of Polymer Science and Engineering, State Key Laboratory of Polymer Materials Engineering, Sichuan University, Chengdu, China

Email: lihongxiang@scu.edu.cn

[3]Function Hub, Advanced Materials Thrust, The Hong Kong University of Science and Technology, Nansha Guangzhou, 511400 China

[4]Equal contribution

Characterization

UV-vis absorption spectra were measured using a Shimadzu UV-2500 recording spectrophotometer. AFM measurements were obtained by using a Dimension Icon AFM (Bruker) in a tapping mode.

GIWAXS: GIWAXS data were obtained at 1W1A Diffuse X-ray Scattering Station, Beijing Synchrotron Radiation Facility (BSRF-1W1A). The monochromatic of the light source was 1.54 Å. The data were recorded by using the two-dimensional image plate detector of Eiger 2M from Dectris, Switzerland. The sample to detector distance (SDD) was set to 110 mm for GIWAXS measurement.

In situ GIWAXS/GISAXS: In-situ spin-coating GIWAXS/GISAXS experiments are conducted using Synchrotron in-situ micro-controlled temperature spin coater, which was jointly developed by Sichuan University, Beijing Synchrotron Radiation Facility, and Beijing Zhongke Wanyuan Technology Co., Ltd. It allows for remote solution dripping, remote speed adjustment, and remote temperature control, it can be used in different types of atmospheres with adjustable gas flow, enabling in-situ GIWAXS/GISAXS monitoring. The in situ GIWAXS/GISAXS data were obtained at beamline BL02U2 and BL6B1 of Shanghai Synchrotron Radiation Facility (SSRF). The monochromatic of the light source was 1.24 Å. The data were recorded by using the two-dimensional image plate detector of Pilatus 2M from Dectris, Switzerland, The sample to detector distance was set to 150 mm and 2100 mm for in situ GIWAXS and GISAXS measurement. The transformation to q-space, radial cuts for the in-plane and out-of-plane analysis and azimuthal cuts for the orientation analysis were processed by GIWAXS-tools and SGTools.

GISAXS fitting

The GISAXS 1D profiles were analyzed using a universal model described by Equation (1) and (2) and fitted to the data using SasView (Version 5.04) software.

$$\mathrm{I}(q) = \frac{A_1}{[1+(q\xi)^2]^2} + A_2 \ \langle \mathrm{P(q,R)} \rangle \ \mathrm{S(q,R,\eta,D)} + \mathrm{B} \tag{1}$$

$$S(q) = 1 + \frac{\sin[(D-1)\tan^{-1}(q\eta)]b}{(qR)^D} \frac{D\Gamma(D-1)}{[1+\frac{1}{(q\eta)^2}]^{(D-1)/2}} \quad (2)$$

In this model, A1, A2, and B are independent fitting parameters, and q represents the scattering wave vector. The first term of the model comprises the average correlation length ξ of the PM6 domain and the Debye-Anderson-Brumberger (DAB) term, while the second term accounts for the contribution from PY-IT fractal-like aggregations. The parameters in the model include R (the mean spherical radius of the primary PY-IT particles), P(q, R) (the form factor of the PY-IT), S(q, R, η, D) (the fractal structure factor describing interactions in the fractal-like aggregation system), η (the correlation length of the fractal-like structure), and D (the fractal dimension of the network). Equation (3) was utilized to determine the average domain size based on the Guinier radius of the fractal-like network, R_g.

$$R_g = \eta\sqrt{\frac{D(D+1)}{2}} \quad 3$$

SCLC Measurements

The electron and hole mobility were measured by using the method of space-charge limited current (SCLC) for electron-only devices with the structure of ITO/ZnO/active layer/PFN-Br-MA/Ag and hole-only devices with the structure of ITO/PEDOT:PSS-TA/active layers/MoO_x/Ag. The charge carrier mobility was determined by fitting the dark current to the model of a single carrier SCLC according to the equation: $J = 9\varepsilon_0\varepsilon_r\mu V^2/8d^3$, where J is the current density, d is the film thickness of the active layer, μ is the charge carrier mobility, ε_r is the relative dielectric constant of the transport medium, and ε_0 is the permittivity of free space. $V = V_{app} - V_{bi}$, where V_{app} is the applied voltage, V_{bi} is the offset voltage. The charge carrier mobility was calculated from the slope of the $J^{1/2} \sim V$ curves. The thickness of target layer is well controlled identical to that of PV's active layer.

Transient Absorption Spectroscopy

Transient absorption spectroscopy (TAS) was measured with an amplified Ti:sapphire femtosecond laser (800 nm wavelength, 50 fs, 1 kHz repetition; Coherent Libra) and a

Helios pump/probe setup (Ultrafast Systems). The 400 nm pump pulses with a pump fluence of 0.5 or < 3 μJ/cm^2 were obtained by frequency doubling the 800 nm fundamental regenerative amplifier output. The white-light continuum probe pulses were generated by focusing a small portion of the regenerative amplifier's fundamental 800 nm laser pulses into a 2 mm sapphire crystal.

Electroluminescence spectroscopy

Electroluminescence (EL) spectroscopy has been an extremely valuable technique for understanding intermolecular charge transfer states (CTS) in OSCs. It operates by injecting electrons/holes into the OSC device which bimolecularly recombines, acting similarly to light emitting diodes (LED). Interestingly, an emission distinctly different than the emission of the donor and acceptor components have been previously observed, suggestive of an additional quantum state. This state is understood as the CTS existing through the molecular interface formed by the donor and acceptor molecules. Further, several studies concluded that such interfaces are the origin (or at least the dominant precursor) of photogenerated free charges (i.e., electron and hole polarons). For quantitative values corresponding to the property of CTS, the reduced EL (*rEL*) can be fitted through Gaussian functions based on the Marcus theory of electron transfer. A more detailed explanation and quantum-mechanical derivation can be found elsewhere.

$$rEL = \frac{f}{\sqrt{4\pi\lambda_{RO}\Phi_T}} exp\left(\frac{-(E_{CTS} - \lambda_{RO} - E)^2}{4\lambda_{RO}\Phi_T}\right)$$

Here, *rEL* is *EL/E* where *E* is the photon energy (eV), *f* is a pre-exponential factor, λ_{RO} is CTS reorganization energy (eV), Φ_T is thermal energy (eV), and E_{CTS} is the CTS energy (eV). It must be noted that further division of *rEL* by E^2 is typically necessary for Jacobian correction, depending on the type of spectrometer used. It is also worthy to deconvolute the blend EL spectra and disentangle contributions from CTS and acceptor singlets.

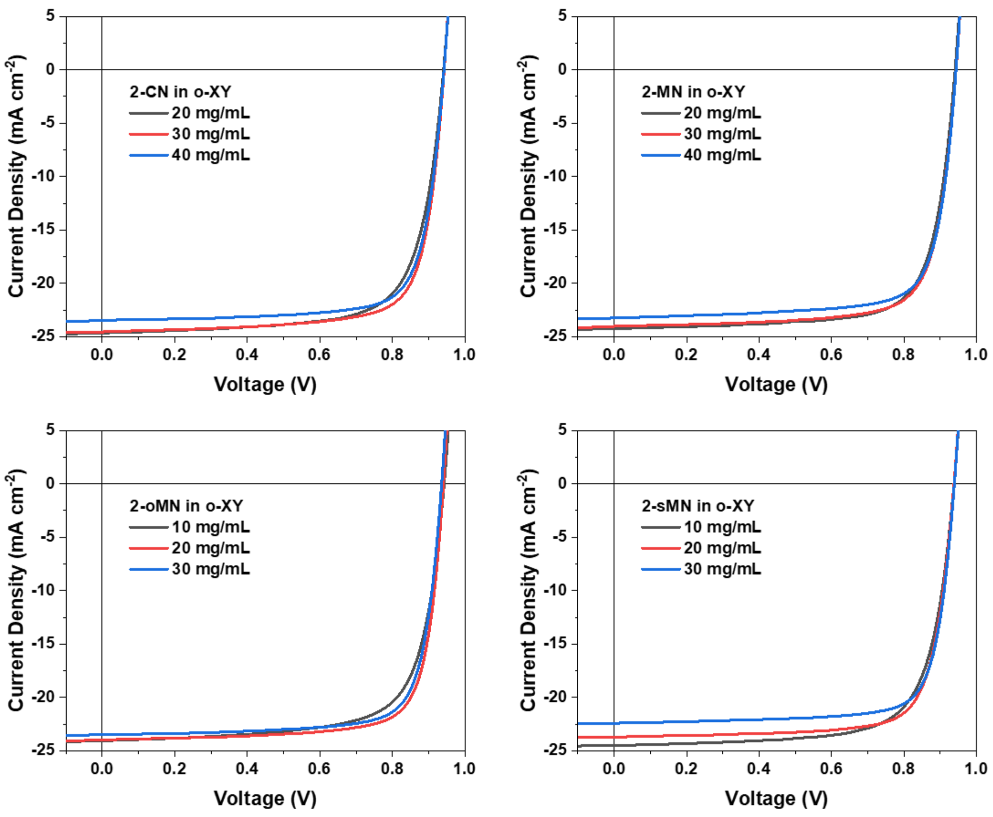

Figure S1. *J-V* characteristics of all additive treated blends.

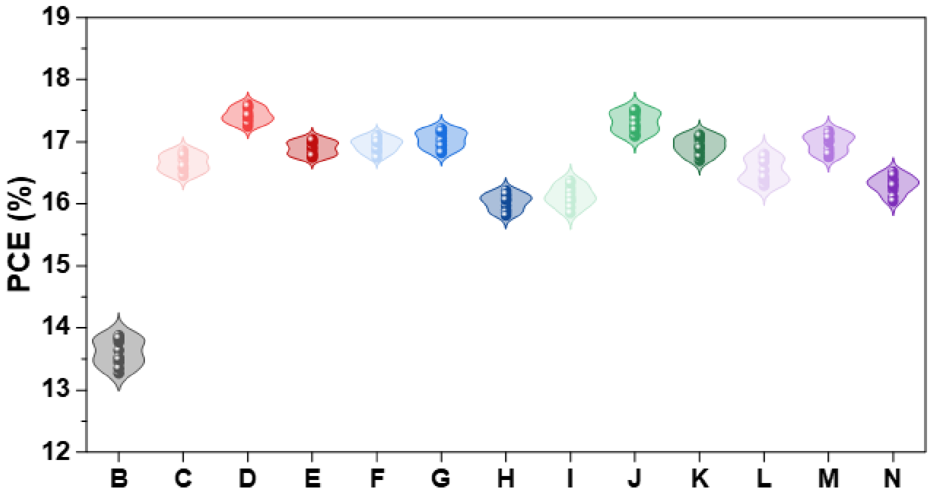

Figure S2. Normal distribution of device efficiencies on all PM6:PY-IT based devices: [B] w/o, [C] 20 mg/ml 2-CN, [D] 30 mg/ml 2-CN, [E] 40 mg/ml 2-CN, [F] 20 mg/ml 2-MN, [G] 30 mg/ml 2-MN, [H] 40 mg/ml 2-MN, [I] 10 mg/ml 2-oMN, [J] 20 mg/ml 2-oMN, [K] 30 mg/ml 2-oMN, [L] 10 mg/ml 2-sMN, [M] 20 mg/ml 2-sMN, [N] 30 mg/ml 2-sMN.

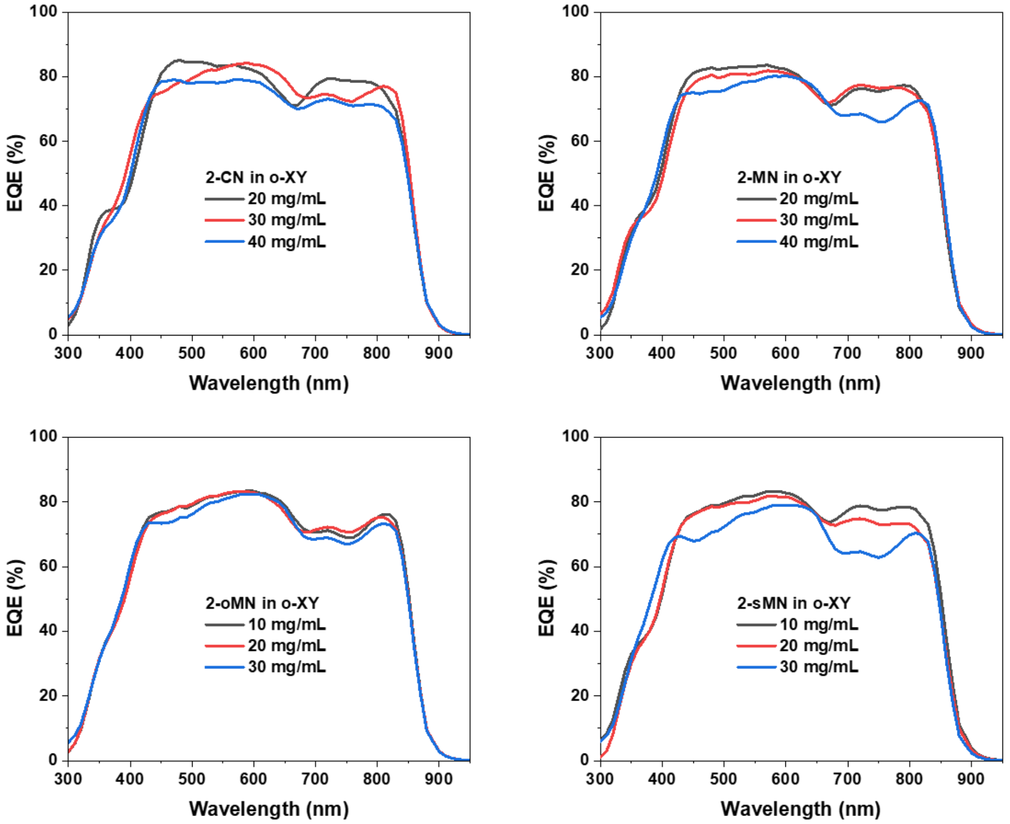

Figure S3. EQE spectra of all additive treated blends.

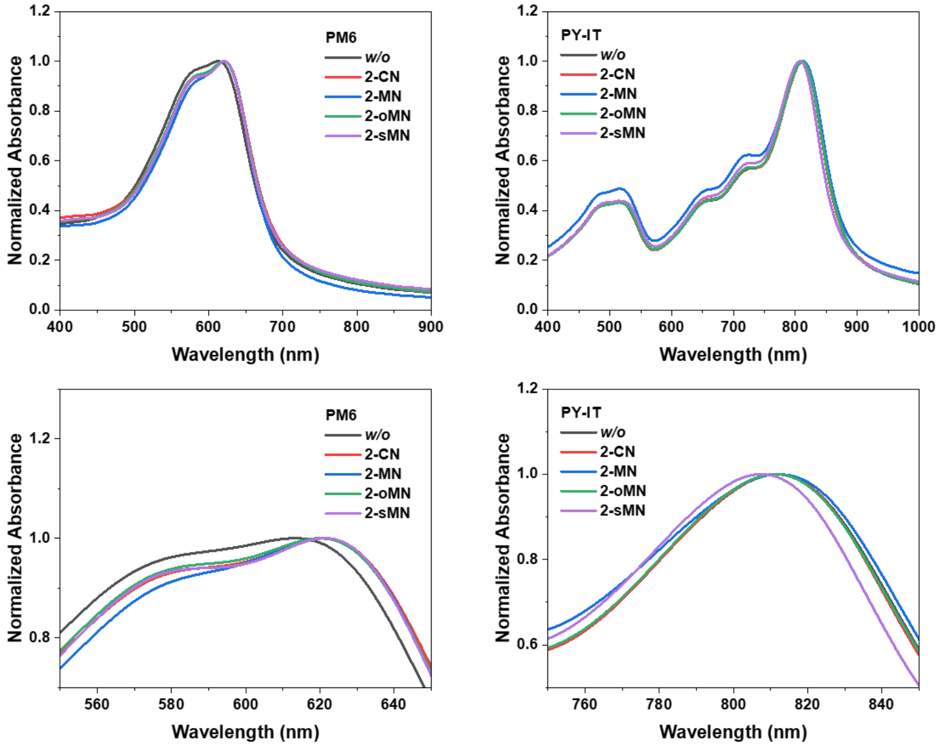

Figure S4. Normalized absorption profiles of neat PM6/PY-IT films treated by different procedures.

Table S1. Device performances.

PM6:PY-IT	V_{OC} (V)	J_{SC} (mA cm^{-2})	*FF* (%)	PCE (%)
w/o	0.946	24.09/23.21	60.9	13.88 (13.60±0.22)
2-CN				
20 mg/ml	0.941	24.66/23.74	72.6	16.85 (16.65±0.14)
30 mg/ml	0.942	24.55/23.64	76.1	17.60 (17.42±0.12)
40 mg/ml	0.942	23.49/22.55	77.0	17.04 (16.89±0.11)
2-MN				
20 mg/ml	0.941	24.26/23.53	74.9	17.10 (16.94±0.12)
30 mg/ml	0.944	24.04/23.31	76.0	17.21 (17.03±0.14)
40 mg/ml	0.944	23.26/22.47	76.6	16.21 (16.12±0.17)
2-oMN				
10 mg/ml	0.942	24.07/23.34	72.2	16.37 (16.02±0.13)
20 mg/ml	0.941	23.99/23.23	77.6	17.52 (17.32±0.16)
30 mg/ml	0.935	23.49/22.77	77.9	17.11 (16.93±0.14)
2-sMN				
10 mg/ml	0.938	24.54/23.79	73.0	16.80 (16.54±0.19)
20 mg/ml	0.938	23.71/22.99	77.2	17.17 (16.98±0.15)
30 mg/ml	0.939	22.42/21.64	78.4	16.51 (16.30±0.15)

The average values are obtained through 10 independent devices.

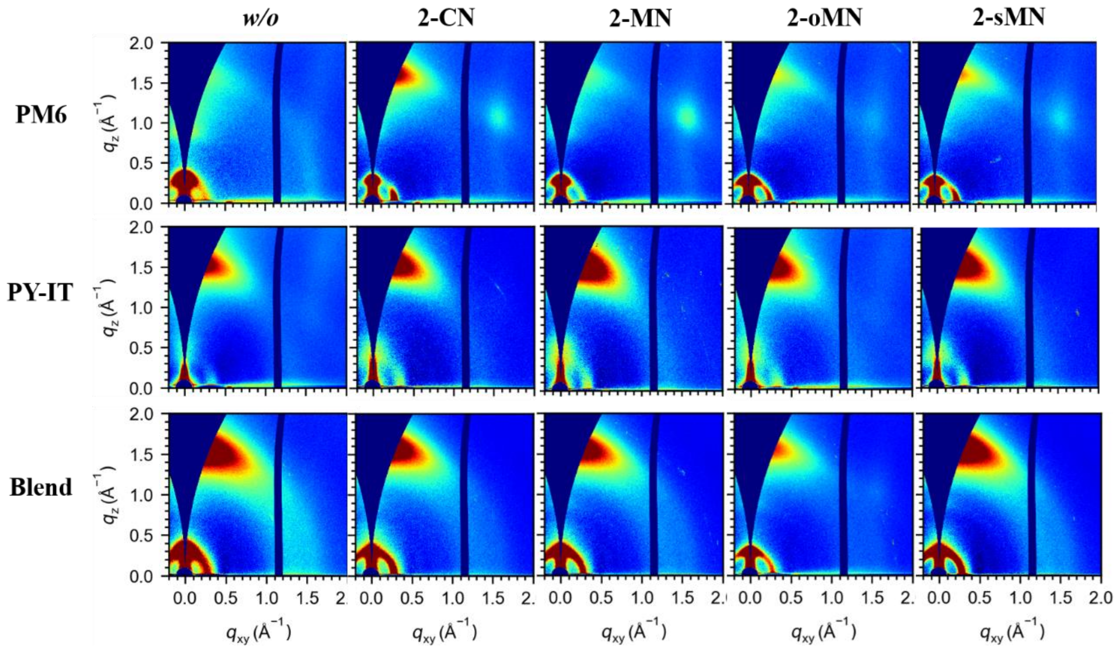

Figure S5. 2D GIWAXS patterns.

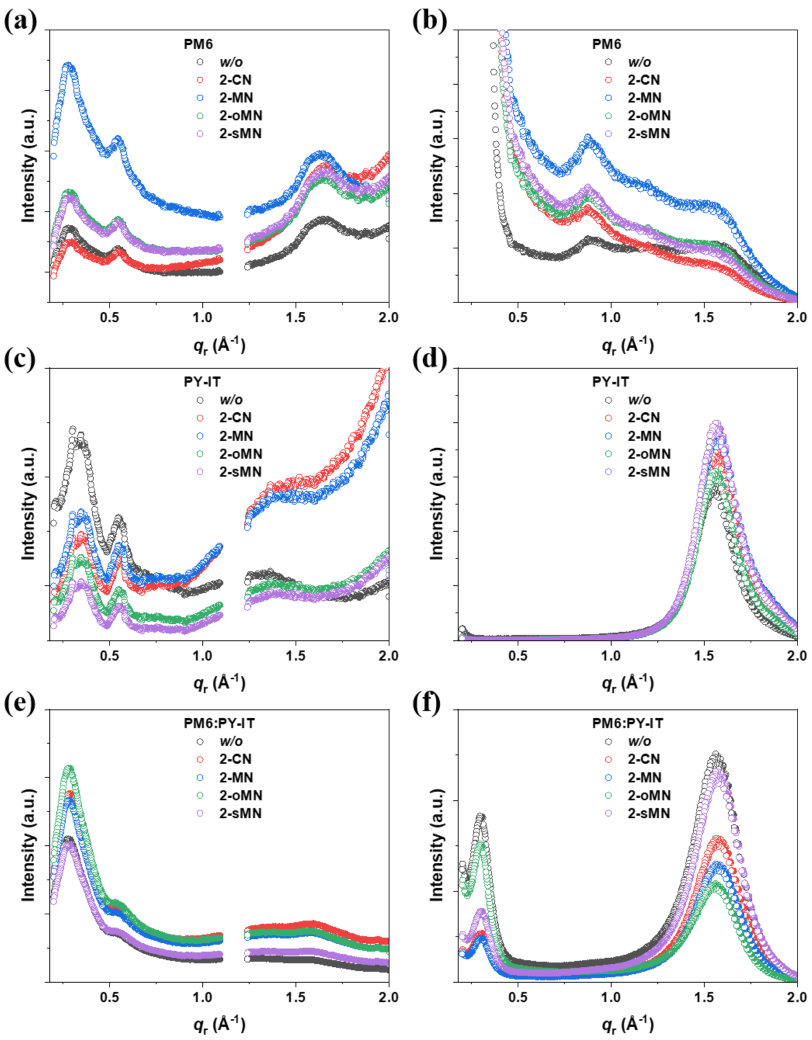

Figure S6. Line-cuts: PM6 films with different from processing on (a) in-plane and (b) out-of-plane directions. PY-IT films with different from processing on (c) in-plane and (d) out-of-plane directions. PM6:PY-IT films with different from processing on (e) in-plane and (f) out-of-plane directions.

Table S2. Calculated parameters for lamellar peaks at IP direction.

Systems	Peak position (Å^{-1})	d-spacing (Å)	CCL (Å)
**PM6**			
w/o	0.275; 0.367; 0.549	22.81; 17.10; 11.45	55.29; 30.50; 63.35
2-CN	0.282; 0.364; 0.549	22.27; 17.24; 11.45	69.30; 37.95; 62.14
2-MN	0.274; 0.331; 0.549	22.91; 19.01; 11.45	57.20; 22.25; 68.75
2-oMN	0.276; 0.366; 0.548	22.78; 17.15; 11.46	56.24; 30.67; 65.04
2-sMN	0.277; 0.364; 0.548	22.71; 17.26; 11.45	57.52; 30.38; 64.44
**PY-IT**			
w/o	0.326; 0.553	19.30; 11.36	28.86; 70.33

2-CN	0.346; 0.554	18.18; 11.34	46.77; 87.75
2-MN	0.342; 0.553	18.35; 11.36	42.18; 84.51
2-oMN	0.337; 0.552	18.66; 11.37	35.85; 78.24
2-sMN	0.339; 0.553	18.55; 11.37	37.76; 80.38
**PM6:PY-IT**			
w/o	0.277; 0.397; 0.517	22.65; 15.82; 12.14	36.00; 55.08; 26.99
2-CN	0.279; 0.394; 0.533	22.52; 15.96; 11.79	39.28; 50.02; 34.08
2-MN	0.279; 0.395; 0.528	22.50; 15.90; 11.91	37.91; 52.39; 29.61
2-oMN	0.279; 0.395; 0.528	22.53; 15.90; 11.89	38.38; 51.94; 31.94
2-sMN	0.279; 0.396; 0.526	22.55; 15.86; 11.95	37.15; 52.63; 29.83

Table S3. Calculated parameters for π-π peaks at IP direction.

**Systems**	Peak position ($Å^{-1}$)	d-spacing (Å)	CCL (Å)
**PM6**			
w/o	1.617; 1.807	3.89; 3.48	27.43; 11.04
2-CN	1.630; 2.038	3.85; 3.08	22.92; 11.78
2-MN	1.614; 1.784	3.85; 3.52	26.77; 18.93
2-oMN	1.624; 1.960	3.87; 3.21	24.39; 11.46
2-sMN	1.624; 1.996	3.87; 3.15	25.73; 7.28
**PM6:PY-IT**			
w/o	1.303; 1.598	4.82; 3.93	11.75; 30.85
2-CN	1.354; 1.598	4.64; 3.93	19.27; 26.60
2-MN	1.344; 1.600	4.67; 3.93	16.94; 26.70
2-oMN	1.349; 1.595	4.66; 3.94	14.83; 32.40
2-sMN	1.340; 1.590	4.69; 3.95	14.83; 34.16

Table S4. Calculated parameters for lamellar peaks at OOP direction.

**Systems**	Peak position ($Å^{-1}$)	d-spacing (Å)	CCL (Å)
**PM6**			
w/o	0.266; 0.288	23.61; 21.79	36.06; 102.97
2-CN	0.259; 0.288	24.22; 21.84	40.46; 102.85
2-MN	0.264; 0.288	23.79; 21.81	41.98; 103.85
2-oMN	0.261; 0.288	24.04; 21.82	41.03; 102.87
2-sMN	0.260; 0.288	24.14; 21.83	40.61; 102.93
**PM6:PY-IT**			
w/o	0.302	20.83	61.91

2-CN	0.303	20.74	63.78
2-MN	0.304	20.68	64.66
2-oMN	0.302	20.84	68.84
2-sMN	0.305	20.63	66.76

Table S5. Calculated parameters for π-π peaks at OOP direction.

**Systems**	Peak position ($Å^{-1}$)	d-spacing (Å)	CCL (Å)
**PY-IT**			
w/o	1.550; 1.558	4.05; 4.03	29.76; 13.81
2-CN	1.560; 1.619	4.03; 3.88	28.88; 12.49
2-MN	1.562; 1.630	4.02; 3.86	28.90; 12.45
2-oMN	1.556; 1.596	4.04; 3.94	29.32; 13.10
2-sMN	1.560; 1.619	4.03; 3.88	29.01; 12.60
**PM6:PY-IT**			
w/o	1.461; 1.569	4.30; 4.00	8.12; 22.66
2-CN	1.497; 1.575	4.20; 3.99	8.03; 22.67
2-MN	1.493; 1.575	4.20; 3.99	7.99; 22.64
2-oMN	1.426; 1.563	4.41; 4.02	8.41; 22.79
2-sMN	1.498; 1.576	4.19; 3.99	7.93; 22.58

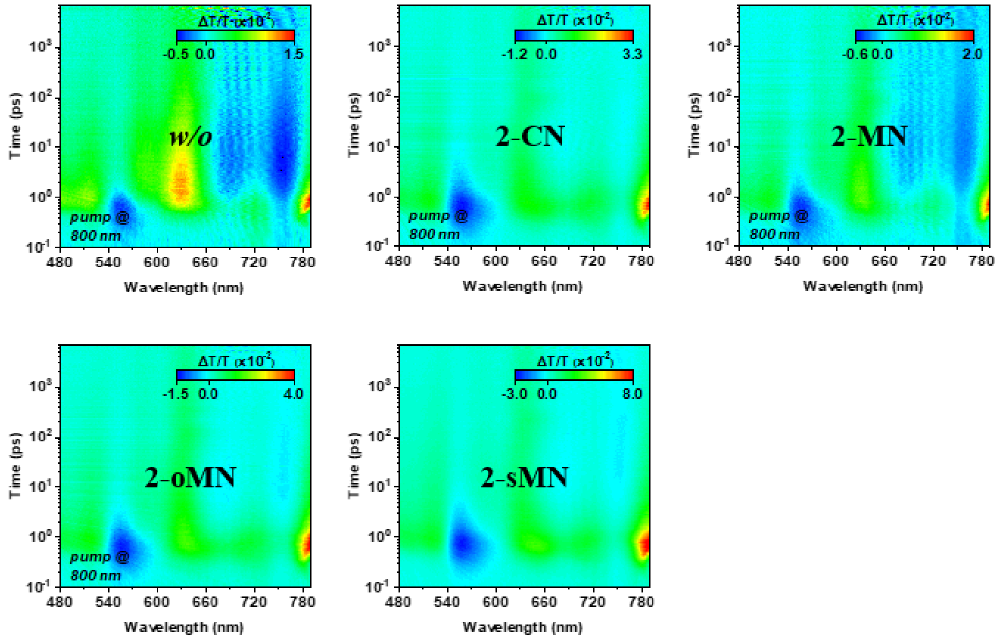

Figure S7. 2D contour maps of TAS measurement results of PM6:PY-IT blend films.

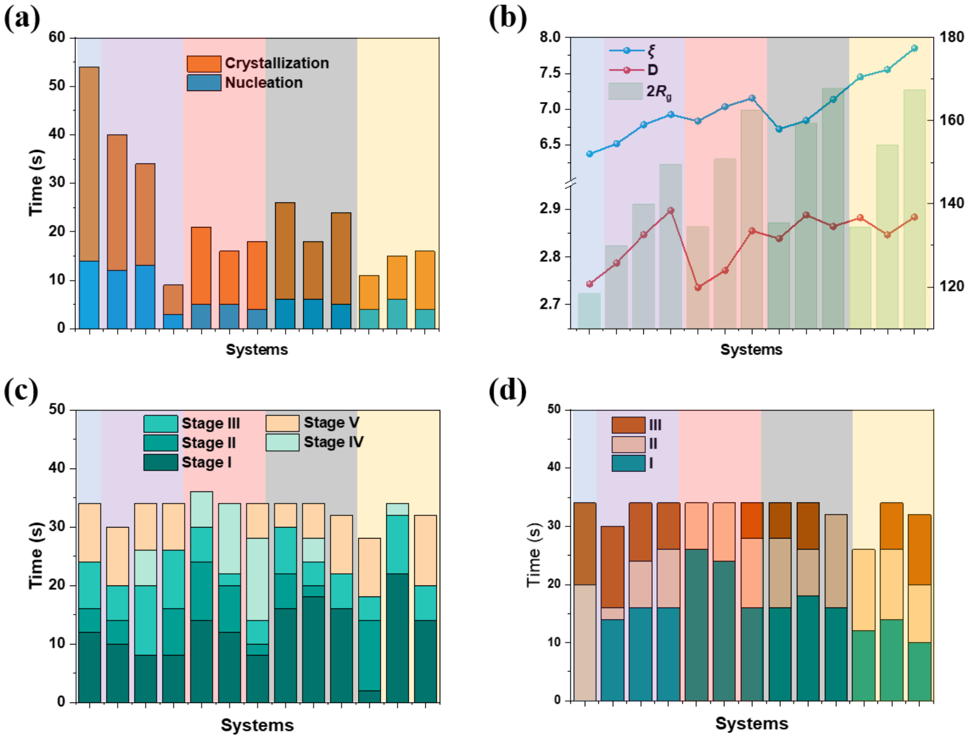

Figure S8. (a) In-situ crystallization processing screening. (b) Steady-state domain size results. (c) Donor phase expansion stages. (d) Acceptor phase expansion stages.

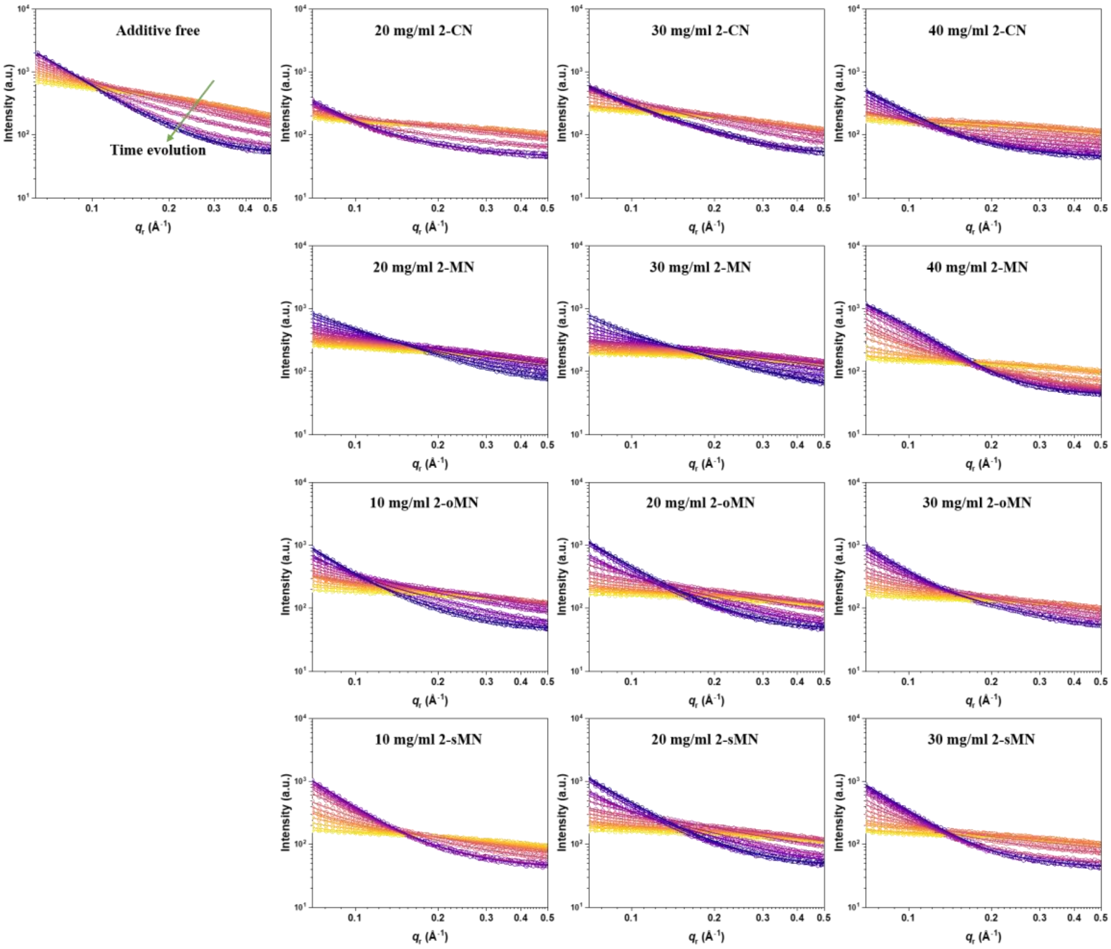

Figure S9. In-plane intensity profiles and fitting lines for all systems at typical times.

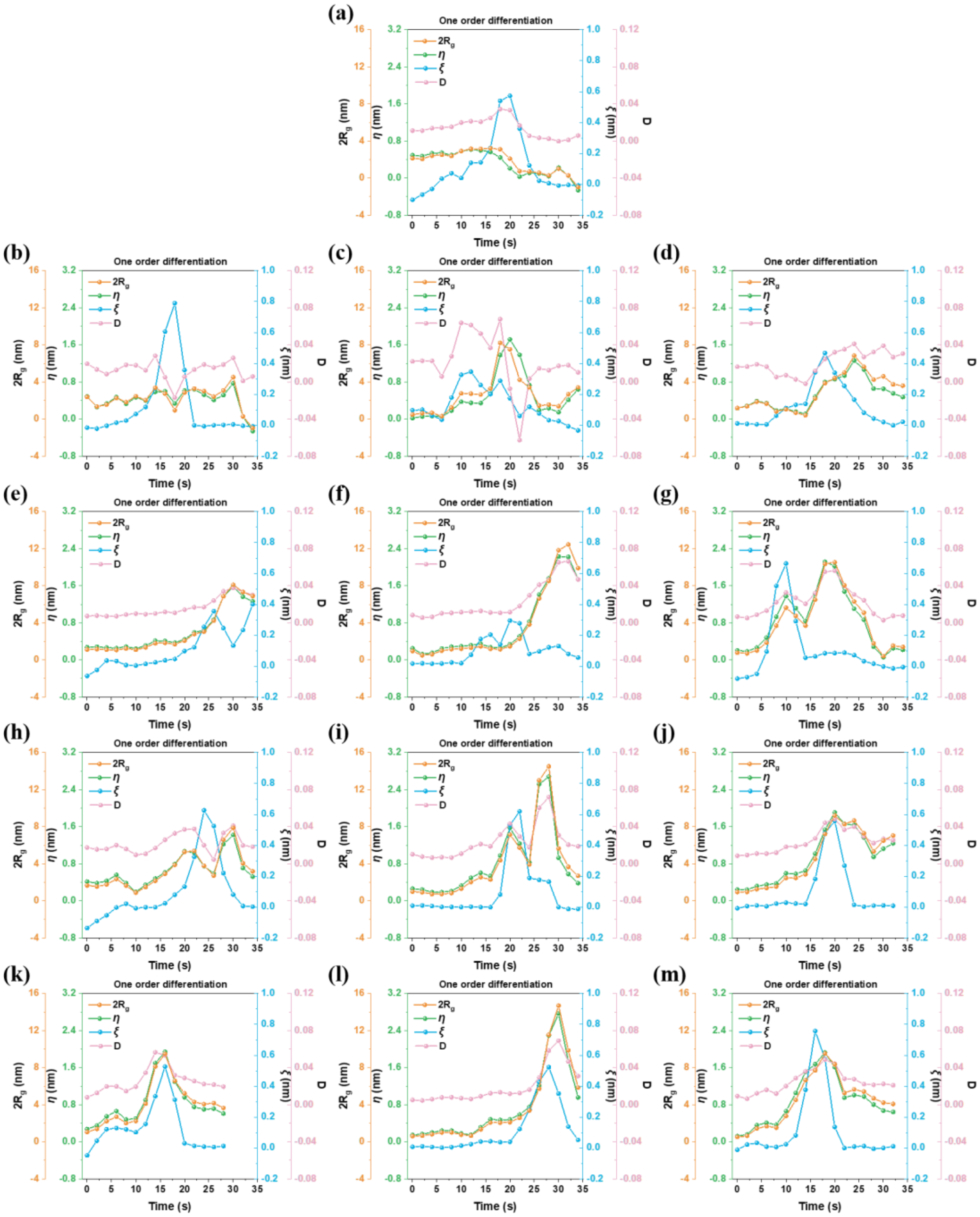

Figure S10. One order differentiation on real time crystallization process of PM6:PY-IT films of (a) w/o, (b) 20 mg/ml 2-CN, (c) 30 mg/ml 2-CN, (d) 40 mg/ml 2-CN, (e) 20 mg/ml 2-MN, (f) 30 mg/ml 2-MN, (g) 40 mg/ml 2-MN, (h) 10 mg/ml 2-oMN, (i) 20 mg/ml 2-oMN, (j) 30 mg/ml 2-oMN, (k) 10 mg/ml 2-sMN, (l) 20 mg/ml 2-sMN, (m) 30 mg/ml 2-sMN.

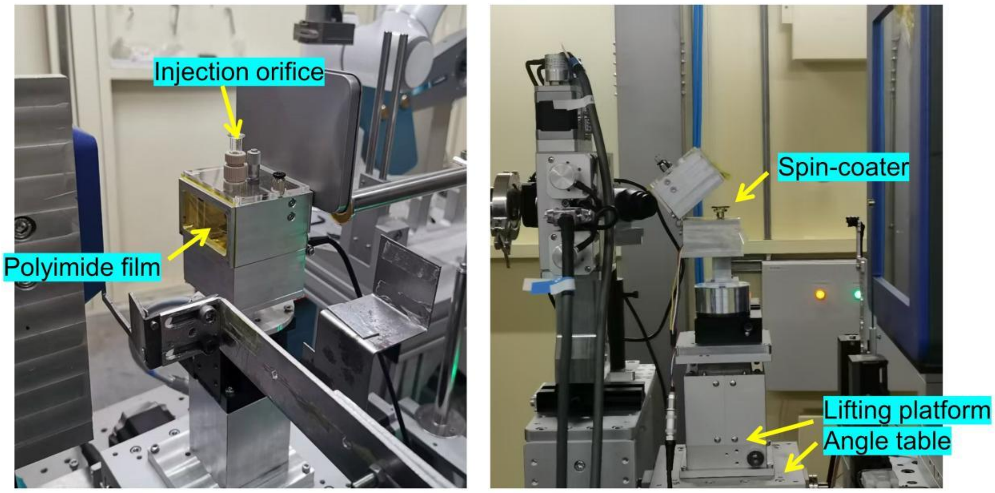

Figure S11: Detailed diagram of the spin-coater.

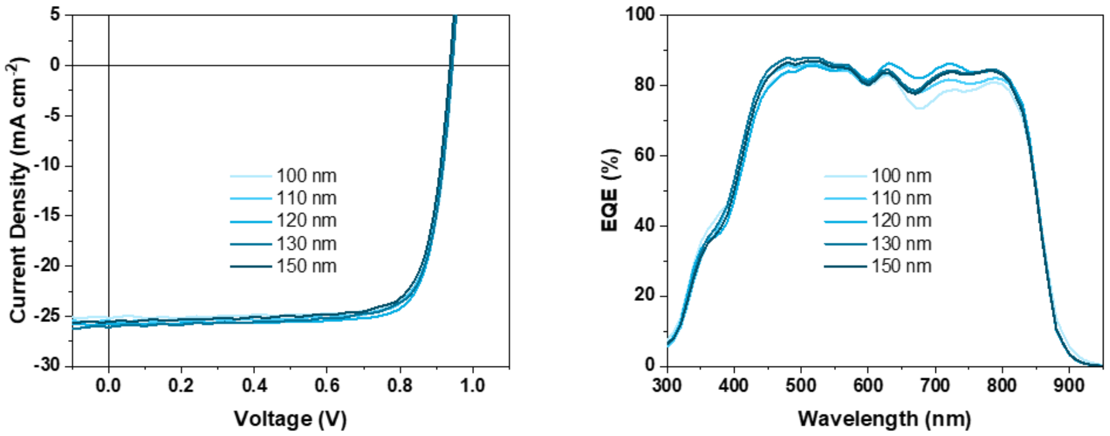

Figure S12. Thickness varied *J-V* characteristics and EQE spectra for ternary all-PSCs.

Table S6. Device performances.

PM6:PBQx-TCl:PY-IT	V_{OC} (V)	J_{SC} (mA cm^{-2})	*FF* (%)	PCE (%)
100 nm	0.944	25.13/24.29	79.2	18.79
110 nm	0.940	25.47/24.65	78.9	18.89
120 nm	0.939	25.81/24.98	79.5	19.27
130 nm	0.944	26.07/25.10	77.1	18.97
150 nm	0.939	25.56/24.77	77.8	18.67

Table S7. Photovoltaic performances of non-halogenated solvent processed all-PSCs

Ref	Solvents	V_{OC} (V)	J_{SC} (mA cm^{-2})	*FF* (%)	PCE (%)
**1**	o-XY+1-MN	0.95	22.10	74.10	15.62

**2**	Tol+CN	0.87	25.35	76.93	17.01
**3**	o-XY+2-oMN	0.95	23.09	77.30	17.03
**4**	Tol+DTT	0.89	25.12	76.64	17.06
**5**	o-XY+2-oMN	0.95	24.07	77.70	17.67
**6**	Tol+CN	0.91	25.7	77.0	18.1
**7**	Tol+CN	0.94	25.25	79.0	18.81
**8**	o-XY+DTT2	0.93	25.95	77.26	18.72
**9**	o-XY+CN	0.915	25.72	78.5	18.47
**10**	o-XY+DMN	0.88	25.23	77.11	17.21
**11**	Tol+CN	0.920	24.3	80.7	18.0
**12**	Tol+2-oMN	0.938	24.61	79.66	18.39
**13**	Tol+CN	0.931	24.5	79.6	18.2
**14**	Tol+CN	0.966	25.16	76.33	18.55
**15**	Tol+CN	0.955	25.16	77.47	18.65
**This work**	o-XY+2-oMN	0.939	25.81	79.5	19.27

Table S8. Photovoltaic performances of large-area all-PSCs

**Ref**	**Area (cm^2)**	**V_{OC} (V)**	**J_{SC} (mA cm^{-2})**	***FF* (%)**	**PCE (%)**
**16**	1	0.911	19.86	62.1	11.24
**17**	1.21	0.908	22.3	68.1	13.81
**18**	1.21	0.939	21.71	72.12	14.70
**19**	1	0.929	22.70	71.79	15.14
**20**	1	0.954	25.43	72.7	17.63
**21**	0.92	0.951	23.32	69.6	15.45
**21**	1	0.958	22.85	68.0	14.90
**This work**	1	0.941	25.85	72.6	17.66

Reference

1. B. Liu, H. Sun, J.-W. Lee, J. Yang, J. Wang, Y. Li, B. Li, M. Xu, Q. Liao, W. Zhang, D. Han, L. Niu, H. Meng, B. J. Kim, X. Guo, *Energy Environ. Sci.* **2021**, 14, 4499.

2. Y. Huang, H. Chen, Q. Fan, Z. Chen, J. Ding, H. Yang, Z. Sun, R. Zhang, W. Chen, C. Yang, F. Gao, Y. Li, *Chin. J. Chem.* **2023**, 41, 1066.

3. J. Song, Y. Li, Y. Cai, R. Zhang, S. Wang, J. Xin, L. Han, D. Wei, W. Ma, F. Gao, Y. Sun, *Matter* **2022**, 5, 4047.

4. K. Hu, C. Zhu, K. Ding, S. Qin, W. Lai, J. Du, J. Zhang, Z. Wei, X. Li, Z. Zhang, L. Meng, H. Ade, Y. Li, *Energy Environ. Sci.* **2022**, 15, 4157.

5. J. Song, C. Li, J. Qiao, C. Liu, Y. Cai, Y. Li, J. Gao, M. H. Jee, X. Hao, H. Y. Woo, Z. Tang, H. Yan, Y. Sun, *Matter* **2023**, 6, 1542.

6. C. Zhao, R. Ma, Y. Hou, L. Zhu, X. Zou, W. Xiong, H. Hu, L. Wang, H. Yu, Y. Wang, G. Zhang, J. Yi, L. Chen, D. Wu, T. Yang, G. Li, M. Qiu, H. Yan, S. Li, G. Zhang, *Adv. Energy Mater.* **2023**, 13, 2300904.

7. R. Ma, H. Li, T. A. Dela Peña, X. Xie, P. W.-K. Fong, Q. Wei, C. Yan, J. Wu, P. Cheng, M. Li, G. Li, *Adv. Mater.* **2024**, 36, 2304632.
8. B. Liu, W. Xu, R. Ma, J.-W. Lee, T. A. Dela Peña, W. Yang, B. Li, M. Li, J. Wu, Y. Wang, C. Zhang, J. Yang, J. Wang, S. Ning, Z. Wang, J. Li, H. Wang, G. Li, B. J. Kim, L. Niu, X. Guo, H. Sun, *Adv. Mater.* **2023**, 35, 2308334.
9. J. Zhang, Q. Zhou, J. Xie, J. Zhao, J. Yu, K. Zhang, T. Jia, F. Huang, Y. Cao, *Advanced Functional Materials* **2024**, 34, 2313722.
10. J. Zhang, Q. Huang, K. Zhang, T. Jia, J. Jing, Y. Chen, Y. Li, Y. Chen, X. Lu, H. Wu, F. Huang, Y. Cao, *Energy Environ. Sci.* **2022**, 15, 4561.
11. J. Wang, Y. Cui, Y. Xu, K. Xian, P. Bi, Z. Chen, K. Zhou, L. Ma, T. Zhang, Y. Yang, Y. Zu, H. Yao, X. Hao, L. Ye, J. Hou, *Adv. Mater.* **2022**, 34, 2205009.
12. P. Bi, T. Zhang, Y. Cui, J. Wang, J. Qiao, K. Xian, X. W. Chua, Z. Chen, W. P. Goh, L. Ye, X. Hao, J. Hou, L. Yang, *Adv. Energy Mater.* **2023**, 13, 2302252.
13. L. Ma, Y. Cui, J. Zhang, K. Xian, Z. Chen, K. Zhou, T. Zhang, W. Wang, H. Yao, S. Zhang, X. Hao, L. Ye, J. Hou, *Adv. Mater.* **2023**, 35, 2208926.
14. W. Xu, H. Tian, Y. Ni, Y. Xu, L. Zhang, F. Zhang, S. Wu, S. Young Jeong, T. Huang, X. Du, X. Li, Z. Ma, H. Young Woo, J. Zhang, X. Ma, J. Wang, F. Zhang, *Chem. Eng. J.* **2024**, 493, 152558.
15. T. Chen, X. Zheng, D. Wang, Y. Zhu, Y. Ouyang, J. Xue, M. Wang, S. Wang, W. Ma, C. Zhang, Z. Ma, S. Li, L. Zuo, H. Chen, *Adv. Mater.* **2024**, 36, 2308061.
16. Y.-F. Shen, J. Zhang, C. Tian, D. Qiu, Z. Wei, *Nano Research* **2023**, 16, 13008.
17. D. Chen, S. Liu, B. Huang, J. Oh, F. Wu, J. Liu, C. Yang, L. Chen, Y. Chen, *Small* **2022**, 18, 2200734.
18. J. Liu, J. Deng, Y. Zhu, X. Geng, L. Zhang, S. Y. Jeong, D. Zhou, H. Y. Woo, D. Chen, F. Wu, L. Chen, *Adv. Mater.* **2023**, 35, 2208008.
19. Z. Zhang, Z. Li, P. Wang, H. Chen, K. Ma, Y. Zhang, T. Duan, C. Li, Z. Yao, B. Kan, X. Wan, Y. Chen, *Advanced Functional Materials* **2023**, 33, 2214248.
20. J. Song, C. Li, H. Ma, B. Han, Q. Wang, X. Wang, D. Wei, L. Bu, R. Yang, H. Yan, Y. Sun, *Adv. Mater.* **2024**, n/a, 2406922.
21. T. Liu, T. Yang, R. Ma, L. Zhan, Z. Luo, G. Zhang, Y. Li, K. Gao, Y. Xiao, J. Yu, X. Zou, H. Sun, M. Zhang, T. A. Dela Peña, Z. Xing, H. Liu, X. Li, G. Li, J. Huang, C. Duan, K. S. Wong, X. Lu, X. Guo, F. Gao, H. Chen, F. Huang, Y. Li, Y. Li, Y. Cao, B. Tang, H. Yan, *Joule* **2021**, 5, 914.
