# Supplementary figures and images for: In-situ understanding on the formation of fibrillar morphology in green solvent processed all-polymer solar cells
Source: Natl Sci Rev. 2024 Nov 4;11(12):nwae384. doi: 10.1093/nsr/nwae384 (PMC11629699; doi:10.1093/nsr/nwae384)

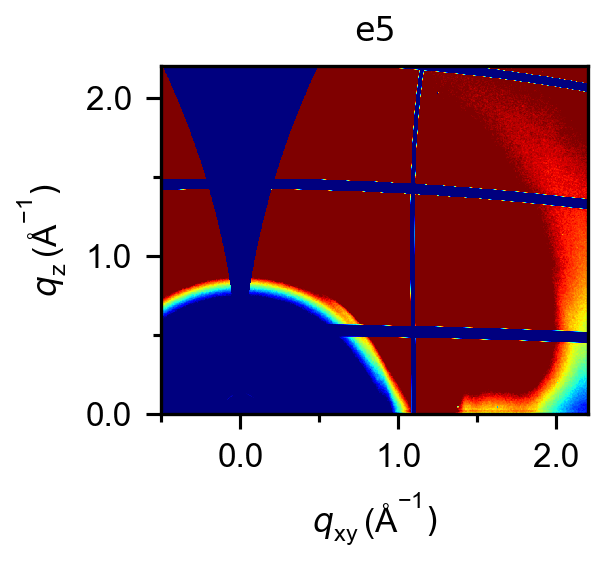

Supplement: nwae384_Supplemental_Files [file nwae384_supplemental_files.zip › In-situ GIWAXS/10mg-2-oMN.gif]

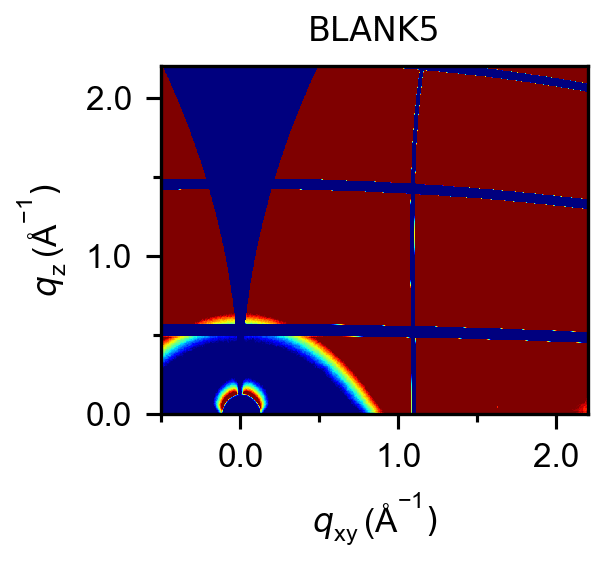

Supplement: nwae384_Supplemental_Files [file nwae384_supplemental_files.zip › In-situ GIWAXS/10mg-2-sMN.gif]

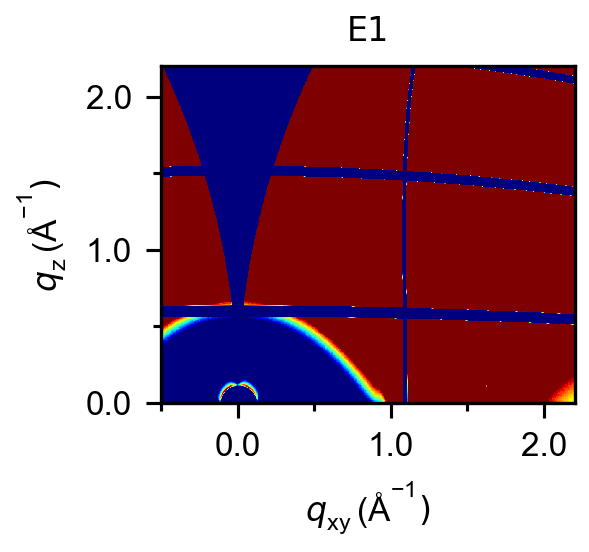

Supplement: nwae384_Supplemental_Files [file nwae384_supplemental_files.zip › In-situ GIWAXS/20mg-2-CN.gif]

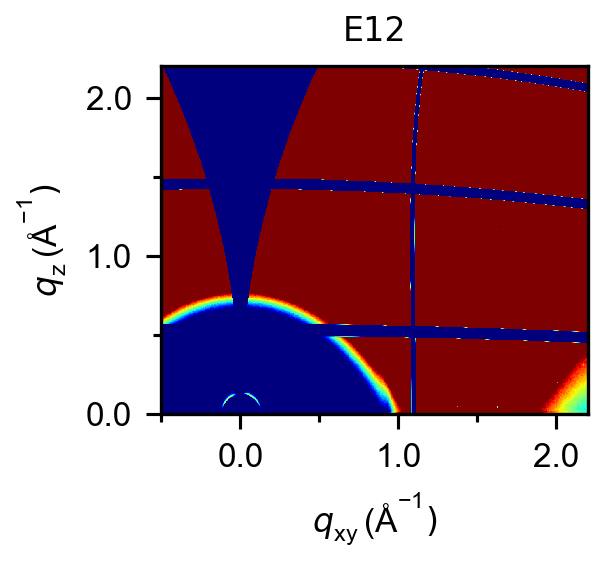

Supplement: nwae384_Supplemental_Files [file nwae384_supplemental_files.zip › In-situ GIWAXS/20mg-2-MN.gif]

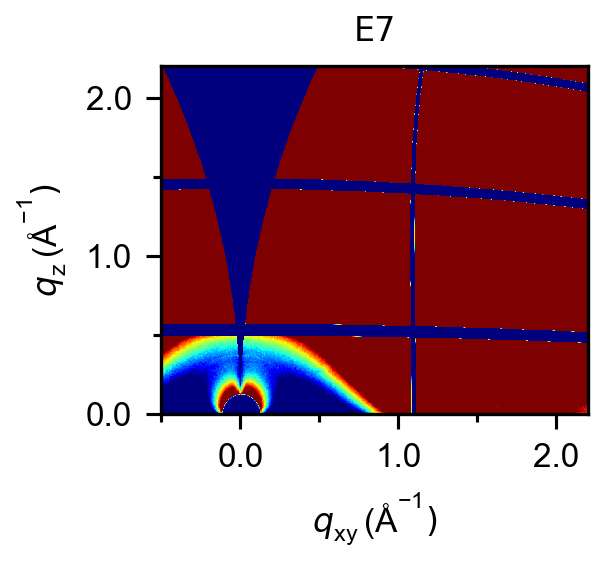

Supplement: nwae384_Supplemental_Files [file nwae384_supplemental_files.zip › In-situ GIWAXS/20mg-2-oMN.gif]

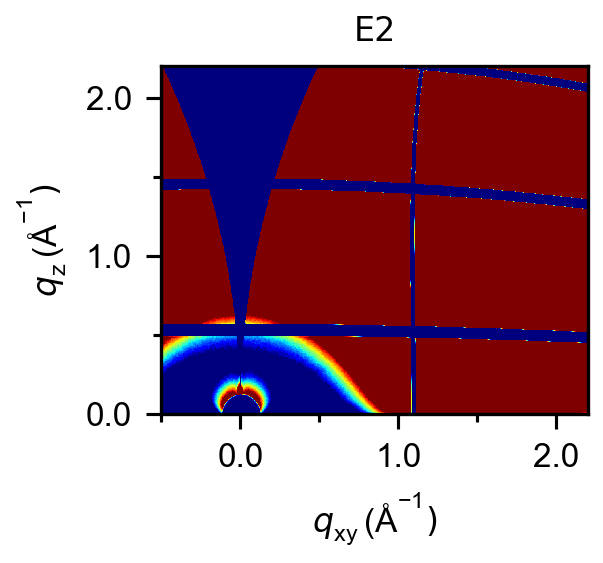

Supplement: nwae384_Supplemental_Files [file nwae384_supplemental_files.zip › In-situ GIWAXS/20mg-2-sMN.gif]

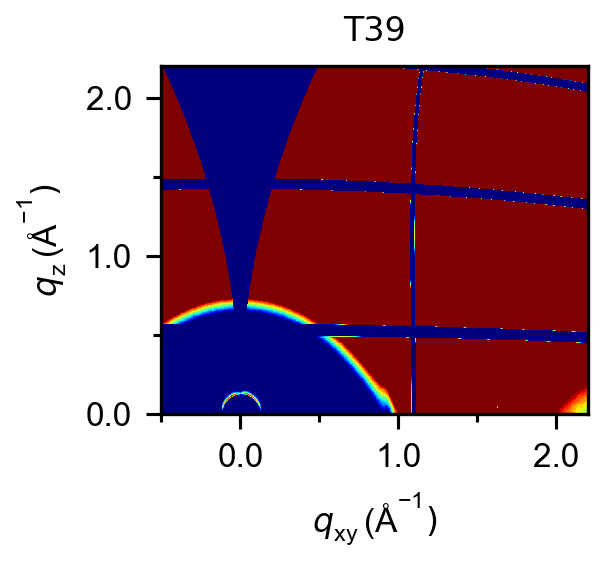

Supplement: nwae384_Supplemental_Files [file nwae384_supplemental_files.zip › In-situ GIWAXS/30mg-2-CN.gif]

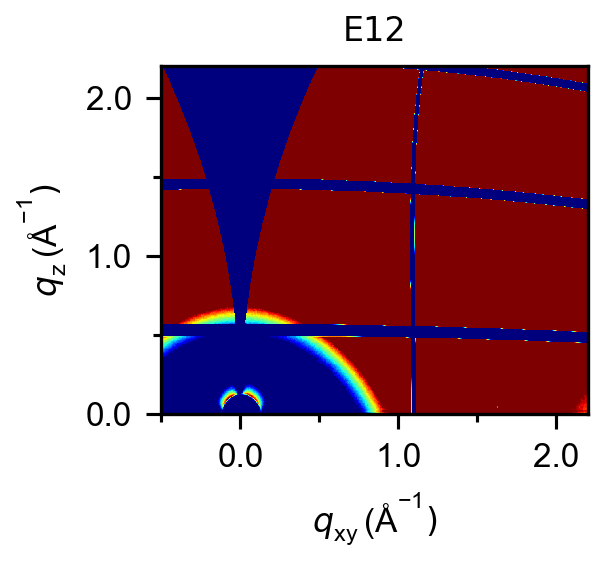

Supplement: nwae384_Supplemental_Files [file nwae384_supplemental_files.zip › In-situ GIWAXS/30mg-2MN.gif]

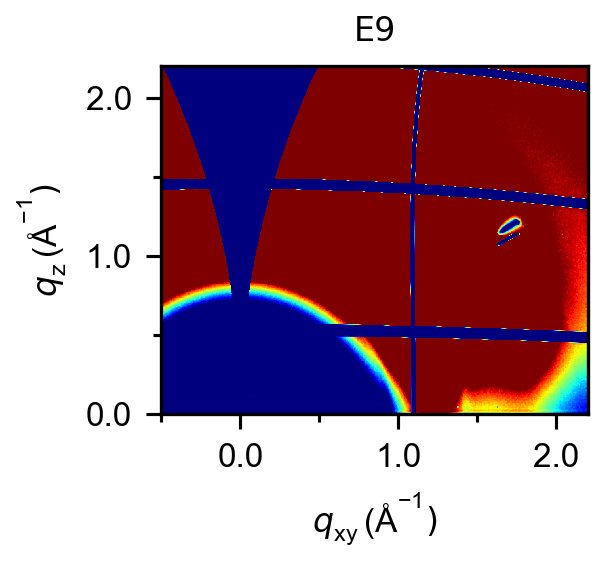

Supplement: nwae384_Supplemental_Files [file nwae384_supplemental_files.zip › In-situ GIWAXS/30mg-2-oMN.gif]

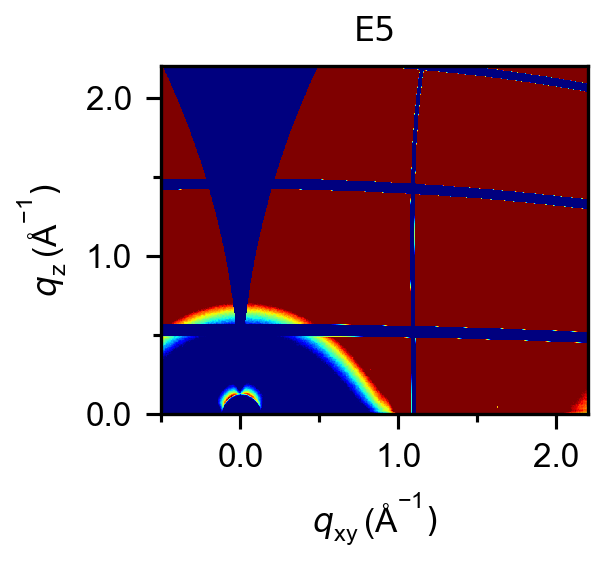

Supplement: nwae384_Supplemental_Files [file nwae384_supplemental_files.zip › In-situ GIWAXS/30mg-2-sMN.gif]

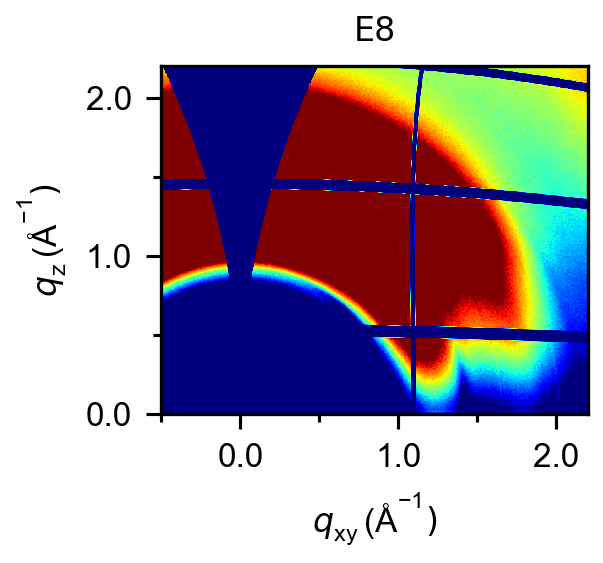

Supplement: nwae384_Supplemental_Files [file nwae384_supplemental_files.zip › In-situ GIWAXS/40mg-2-CN.gif]

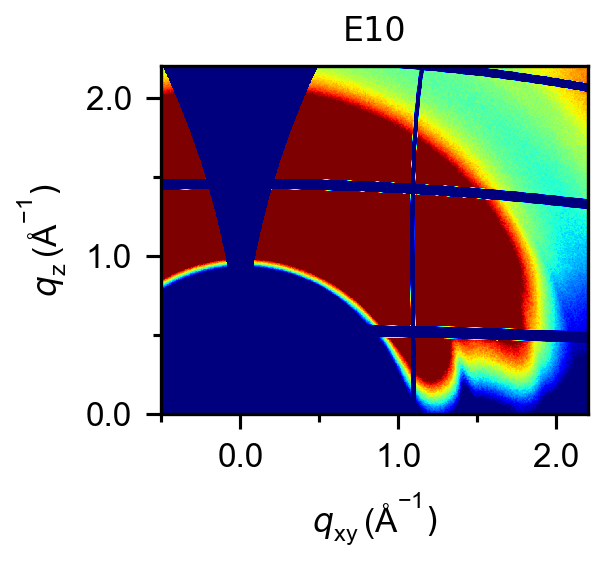

Supplement: nwae384_Supplemental_Files [file nwae384_supplemental_files.zip › In-situ GIWAXS/40mg-2-MN.gif]

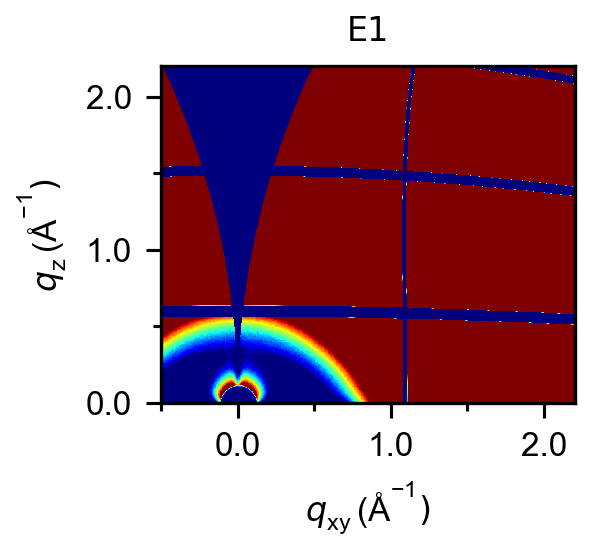

Supplement: nwae384_Supplemental_Files [file nwae384_supplemental_files.zip › In-situ GIWAXS/additive-free.gif]
